# Dynamic mitophagy trajectories hallmark brain aging

**DOI:** 10.1080/15548627.2024.2426115

**Published:** 2024-12-19

**Authors:** Anna Rappe, Thomas G. McWilliams

**Affiliations:** a Translational Stem Cell Biology and Metabolism Program, Faculty of Medicine, Biomedicum Helsinki, University of Helsinki, Helsinki, Finland; b Department of Anatomy, Faculty of Medicine, University of Helsinki, Helsinki, Finland

**Keywords:** Aging, autophagy, brain, mitochondria, mitophagy

## Abstract

Studies using mitophagy reporter mice have established steady-state landscapes of mitochondrial destruction in mammalian tissues, sparking intense interest in basal mitophagy. Yet how basal mitophagy is modified by healthy aging in diverse brain cell types has remained a mystery. We present a comprehensive spatiotemporal analysis of mitophagy and macroautophagy dynamics in the aging mammalian brain, reporting critical region- and cell-specific turnover trajectories in a longitudinal study. We demonstrate that the physiological regulation of mitophagy in the mammalian brain is cell-specific, dynamic and complex. Mitophagy increases significantly in the cerebellum and hippocampus during midlife, while remaining unchanged in the prefrontal cortex (PFC). Conversely, macroautophagy decreases in the hippocampus and PFC, but remains stable in the cerebellum. We also describe emergent lysosomal heterogeneity, with subsets of differential acidified lysosomes accumulating in the aging brain. We further establish midlife as a critical inflection point for autophagy regulation, which may be important for region-specific vulnerability and resilience to aging. By mapping *in* *vivo* autophagy dynamics at the single cell level within projection neurons, interneurons and microglia, to astrocytes and secretory cells, we provide a new framework for understanding brain aging and offer potential targets and timepoints for further study and intervention in neurodegenerative diseases.

The brain and mind are subject to significant alterations during aging, which is the critical risk factor for multiple neurological disorders. Aberrant autophagy is a key hallmark of aging. A general decline in autophagy is seen in short-lived organisms such as yeast, worms, flies, and some human cell models. These tractable paradigms have provided substantial insights into the molecular basis of aging. Yet short- and long-lived organisms faced very different evolutionary selection pressures. Organisms such as *Drosophila* (e.g. 30- to 70-day lifespan) prioritize rapid tissue development and early reproduction at the expense of longevity. Conversely, longer-lived species such as *Mus musculus* (2–3-year lifespan) must balance their post-reproductive lifespan with the energetic demands of somatic maintenance and longevity. These differences highlight distinct survival strategies that have been shaped by natural selection. Scaling mechanistic insights to the complexity of the mammalian brain remains challenging.

The advent of sophisticated mouse models and novel patient cohorts has established the physiological and clinical relevance of basal autophagy. However, significant challenges remain. Our spatiotemporal understanding of autophagy pathways in healthy brain aging remains extremely limited. This knowledge gap is critical because future autophagy-based therapeutics must be accurately profiled with an understanding of when, where and why they alter basal turnover in the brain at the single-cell level. Overcoming these challenges will be critical to the developing precision autophagy medicines and interventions targeting age-related neurodegeneration, where therapeutic failure rates exceed 99%.

We profiled the spatiotemporal dynamics of mitophagy and macroautophagy in mammalian brain aging through a longitudinal study spanning 3–26 months (approximating 20–69 human years, JAX aging guidelines) [1]. Using the well-characterized *mito*-QC and *auto*-QC reporter mouse models ensured an internally controlled design, with consistent genetic backgrounds and distinct reporter patterns *in vivo*. Our analysis revealed that autophagy pathways do not decline uniformly in the mammalian brain. Instead, they are dynamically regulated and show high regional and cell-type specificity with distinct fluctuation patterns. For example, in the cerebellum – responsible for fine motor control – mitophagy increases significantly with age, while macroautophagy remains stable. In contrast, the PFC exhibits unaltered mitophagy but a robust decrease in autophagy. In the hippocampus, which regulates memory, mitophagy increases in midlife and then declines significantly, while macroautophagy shows a gradual decline throughout aging. Non-neuronal cells, like glial and secretory cells, show region-specific differences. The choroid plexus, which produces cerebrospinal fluid, shows a modest increase in mitophagy but no change in macroautophagy, despite having the highest basal level of macroautophagy of all regions examined.

We also found robust remodeling of the endolysosomal network during aging, characterized by increased lysosomal heterogeneity (Figure 1). Aged brains exhibit an increase in double-positive mCherry-GFP puncta localized within LAMP1^+^ LAMP2^+^ CTSB^+^ CTSD^+^ PIP4P1/TMEM55B^+^ endolysosomes. Quantification revealed that differentially acidified lysosomes (DALs) increase with age, and TEM analysis demonstrated continuous remodeling of neuronal endolysosomes throughout life. This suggests that although cargo capture and delivery to lysosomes are broadly maintained in healthy brain aging, lysosomal acidification becomes increasingly compromised. This has important clinical implications, as autophagy-inducing therapeutics may be harmful if lysosomal homeostasis is not preserved. Understanding the basis of lysosomal heterogeneity in the aging brain will be essential for the development of future precision autophagy therapeutics.

**Figure 1.**
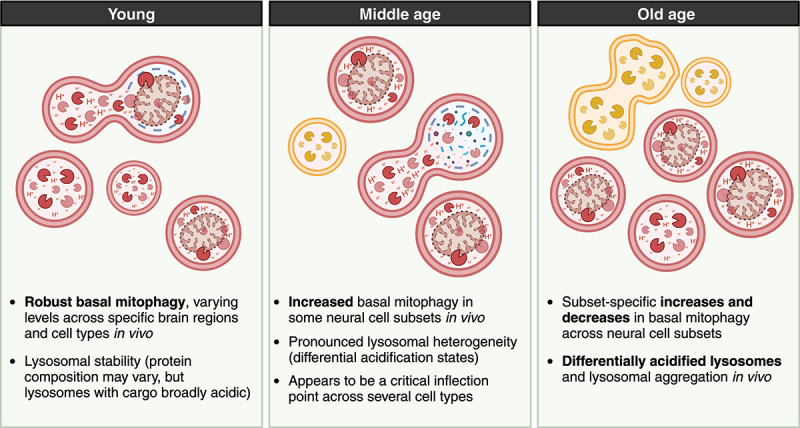
Schematic summarizing broad changes in lysosomal pools throughout brain aging. In some brain regions and neural cell populations, mitophagy increases with age. The increase can either be sustained or be decreased following a midlife peak. Lysosomal heterogeneity is a feature of the mature mammalian brain and is modified by the aging process, characterized by increased lysosomal aggregation and differentially acidified lysosomes.

Importantly, our work identified midlife as a critical turning point for brain autophagy dynamics and lysosomal integrity. For example, cerebellar Purkinje neurons show a robust midlife increase in mitophagy that persists into old age. Similarly, the CA1 and dentate gyrus regions of the hippocampus show increased mitophagy throughout midlife and stabilize later in life at levels comparable to young mice. Interestingly, while autophagy generally declines with age, the accumulation of DALs increases significantly at midlife. Determining whether these inflection points are causally related to age-related cognitive changes in healthy aging is critical.

Our study also reveals autophagy insights into neglected cell populations of outstanding interest in contemporary neurobiology. PVALB (parvalbumin)-positive (PVALB^+^) interneurons are critical modulators of the microcircuitry underpinning memory. PVALB^+^ interneurons in hippocampal CA1 follow similar trajectories in midlife. Although autophagy fluctuations in PVLB^+^ interneurons are more modest than in the broader CA1 region, these neurons show a significant increase in DALs during aging. We also observed increased basal mitophagy in nigrostriatal A9 dopaminergic neurons during healthy aging, while A10 neurons remain unchanged. Understanding the basis of these trajectories may help explain the selective vulnerability of A9 neurons in Parkinson disease, as LRRK2^G2019S^ hyperactive kinase activity suppresses mitophagy in these neurons. Further investigation of DAL accumulation during aging may also provide functional insights into cognitive decline as well as disease-relevant pathomechanisms.

Our findings raise important questions and establish key concepts. Understanding the cell-type and region-specific regulatory mechanisms that control autophagy will be critical for precision targeting of autophagy pathways in neuropathology. Our work provides a blueprint of autophagy pathway trajectories in the healthy aging brain and underscores the need to clarify the stages at which autophagy is altered along the degradative pathway.

As our data show, impaired lysosomal acidification is a hallmark of aging, even when cargo capture and fusion processes remain intact. While mouse models are powerful tools for studying the complexities of brain aging, the dynamics of autophagy in longer-lived mammals remains an open question. Further work is needed to define the characteristics of autophagy in long-lived neural circuits. It will be interesting to explore the interplay between more specialized mitochondrial quality control mechanisms, such as mitochondrially-derived vesicles/MDVs, mitochondrially-derived compartments/MDCs, and mitochondrial inner membrane-derived vesicles/VDIMs. We remain open to the increased granularity that future reporter models might provide, where the turnover of different mitochondrial subcompartments may yield distinct patterns or trajectories *in vivo*. Regardless, our findings are conceptually complemented by the elegant work of Nikoletopoulou and colleagues, whose sophisticated proteomic analysis of degradomes in young and aged mouse brains revealed no major changes in lysosomal mitochondrial protein degradation, consistent with our finding of continuous mitochondrial degradation throughout life. What drives these fluctuations, and how are they relevant to brain aging? Heightened mitophagy in subpopulations such as cerebellar Purkinje neurons may underlie their resilience in common neurodegeneration. However, these connections remain to be fully explored.

## References

[1] Rappe A, Vihinen HA, Suomi F, et al. Longitudinal autophagy profiling of the mammalian brain reveals sustained mitophagy throughout healthy aging. Embo J. 2024 Oct 4; doi: 10.1038/s44318-024-00241-y Epub ahead of print. PMID: 39367235. PMC11612485 39367235

